# Extensive lymphadenectomy may improve survival in node negative oesophageal cancer

**DOI:** 10.1038/s41598-024-53245-3

**Published:** 2024-02-01

**Authors:** Oleksandr Khoma, Steven R. Paredes, Jin-soo Park, Catherine W. Kennedy, Gregory L. Falk

**Affiliations:** 1 School of Medicine, University of Notre Dame, Sydney, NSW Australia; 2 Upper GI Surgery, Concord Repatriation General Hospital, Concord, NSW Australia; 3 Upper GI Surgery, Strathfield Private Hospital, Strathfield, NSW Australia; 4 Upper GI Surgery, Sydney Adventist Hospital, Wahroonga, NSW Australia; 5 Sydney Heartburn Clinic, Lindfield, NSW Australia; 6 Discipline of Surgery, Faculty of Medicine and Health, University of Sydney, Sydney, NSW Australia

**Keywords:** Oesophageal cancer, Surgical oncology

## Abstract

Lymph node metastases are a major prognostic factor in survival of patients with oesophageal cancer. The number of lymph nodes removed during oesophagectomy has been previously proven to be associated with improved survival. The aim of this study was to examine the effect of lymph node harvest on survival specifically in pathologically node negative (pN0) patients with oesophageal cancer. Data were extracted from a prospectively populated single-surgeon database of oesophageal resections for cancer. All consecutive patients with pN0 were included. Patient-specific risk adjusted analysis of overall and disease-free survival was performed to identify the number of lymph nodes associated with improved survival. Inclusion criteria were met by 137 patients (49 squamous cell carcinoma and 88 adenocarcinoma). Adjusted for cancer stage, tumour (histological type, degree of differentiation, lympho-vascular invasion, neo-adjuvant therapy) and patient related factors (age, sex), increased lymph node number was associated with significant improvement in overall (*P* = 0.045) and disease free (*P* = 0.030) survival. Lymph node count ≥ 17 was associated with improved overall and disease-free survival. In this cohort of patients with pathologically node-negative oesophageal cancer, lymph node count of 17 or above was associated with significantly improved survival.

## Introduction

Oesophageal cancer is the sixth leading cause of cancer related death worldwide with rates of adenocarcinoma on the rise^1,2^. Lymph node metastases are common even in early T1 disease and can be present in up to 40% of patients^1^. Extensive lymphadenectomy has been previously shown to improve survival in patients with oesophageal cancer^1,3–5^, however some studies have challenged this paradigm, especially in recipients of neo-adjuvant treatment^3,6^. Current National Comprehensive Cancer Network (NCCN) guidelines recommend minimum of 15 lymph node removal during oesophagectomy^2^. The rationale for adequate lymphadenectomy is that it may provide improved loco-regional disease control as well as allowing for adequate pathological disease staging.

Lymph node count was shown to predict improved survival in patients with gastric cancer who had no lymph node metastasis on final histopathology (pN0)^7^. This could be explained by the stage shift or removal of isolated tumour cell deposits not identified on pathologic examination, or combination effect. In lung cancer, node positivity, and therefore upstaging, was associated with increased number of lymph nodes examined by the pathologist^8^. Another study of patients with proximal urothelial cancers demonstrated improved survival in pN0 patients compared to pNx^9^.

The primary outcome of this study was to examine the effect of increased lymph node harvest in pN0 patients undergoing curative intent oesophagectomy for adenocarcinoma (AC) or squamous cell carcinoma (SCC) of the oesophagus on overall survival. Secondary outcomes included the effect of lymphadenectomy on disease specific survival and subgroup (AC and SCC) and stage specific survival.

## Patients and methods

Data were extracted from a prospectively populated single surgeon database of oesophageal cancers between 1991 and 2016. During this period, a total of 578 patients underwent oesophagectomy for malignancy (excluding gastrointestinal stromal tumours) and this included 416 for AC and 162 for SCC. All consecutive patients who underwent oesophagectomy for AC or SCC and pathologically staged as N0 were included. Surgery was a standardised open Ivor-Lewis oesophagectomy with two field node dissection and adventitial mediastinal dissection.

Number of lymph nodes were reported by a specialist pathologist after examining all lymph nodes cut in a way to allow for maximum surface assessment and immunohistochemistry staining, which was routinely performed.

All patients were routinely followed up post-operatively with a clinical review by the operating surgeon at 6 weeks, 3 months, 6 months, then every 6 months for the first two years, then annually for up to 5 years post-operatively. Routine follow up imaging or endoscopic surveillance was not performed except upon strong patient preference or other clinical indication (for example pre-operative small lung nodule for surveillance). Patients who developed new symptoms or signs concerning for recurrence were promptly evaluated by imaging, endoscopy and biopsies (as was clinically appropriate). Following the 5 year period patients, were followed up on “as required” basis.

The database was approved by the Concord Hospital Human Research Ethics Committee (approval LNR/12/CRGH/248). All participants provided written informed consent to participate in research. Study protocol conforms to the ethical guidelines of the 1975 Declaration of Helsinki.

Overall survival (OS) was analysed using time to death or last follow-up. Disease-free survival (DFS) was measured using time from treatment to tumour recurrence confirmed on imaging or endoscopy, or confirmation of no evidence of disease (NED) or death of the patient.

Patient demographics included sex and age at diagnosis. Tumour characteristics included type (SCC or AC), location, histological grade, presence of lympho-vascular invasion (LVI) and American Joint Committee on Cancer (AJCC) 8th edition stage. The entire cohort was re-staged according to the AJCC 8th edition when it became available by one of the authors (CWK)^10^.

Statistical analyses were performed using SPSS 26 (IBM, New York). A two-sided *P*-value of < 0.05 was considered significant. Initial analysis was performed using < 15 nodes and ≥ 15 nodes as per NCCN guidelines, later analysis was performed to determine if higher number of nodes was associated with improved survival. Subgroup analysis was performed for each histological subtype (AC and SCC) and tumour stage. Using the Kaplan–Meier method and Log Rank test overall survival and disease-free survival between each lymph node group was compared. Potential confounders were adjusted using a Cox proportional hazards regression model. Furthermore, the Mann–Whitney *U* test was used to evaluate the treatment effect of neoadjuvant radiotherapy on number of resected lymph nodes during surgery.

## Results

Table 1 summarises the demographics of the 137 patients included in the study. Most were male (68.6%) and the median age was 66 years (IQR 60–73). The median number of lymph nodes resected during surgery was 17 (IQR 12–25). Most tumours were adenocarcinoma (64%) and less than 10% of patients underwent neoadjuvant radiotherapy. The median follow-up time for the whole cohort was 8.1 years.

**Table 1:** Characteristics of patients with pN0 oesophageal cancer (*n* = 137).

Characteristic	Number of patients	Percentage
Age		
< 65 years	55	40.1
≥ 65 years	82	59.9
Sex		
Female	43	31.4
Male	94	68.6
Histology		
Adenocarcinoma	88	64.2
Squamous cell carcinoma	49	35.8
Tumour location		
Upper oesophagus	5	3.6
Middle oesophagus	28	20.4
Lower oesophagus	56	40.9
Gastro-oesophageal junction	48	35.0
Histological grade		
G1 (well differentiated)	19	13.9
G2 (moderately differentiated)	74	54.0
G3 (poorly differentiated)	44	32.1
Presence of lymphovascular invasion		
No	113	82.5
Yes	24	17.5
Tumour stage (AJCC 8th edition)		
I	64	46.7
II	47	34.3
III	23	16.8
IV	3	2.2
Number of nodes resected		
< 17	66	48.2
≥ 17	71	51.8
Neoadjuvant therapy		
None	69	50.3
Radiotherapy	13	9.5
Chemotherapy	43	31.4
Chemoradiotherapy	12	8.8
Adjuvant chemotherapy		
No	121	88.3
Yes	16	11.7

**Figure 1:**
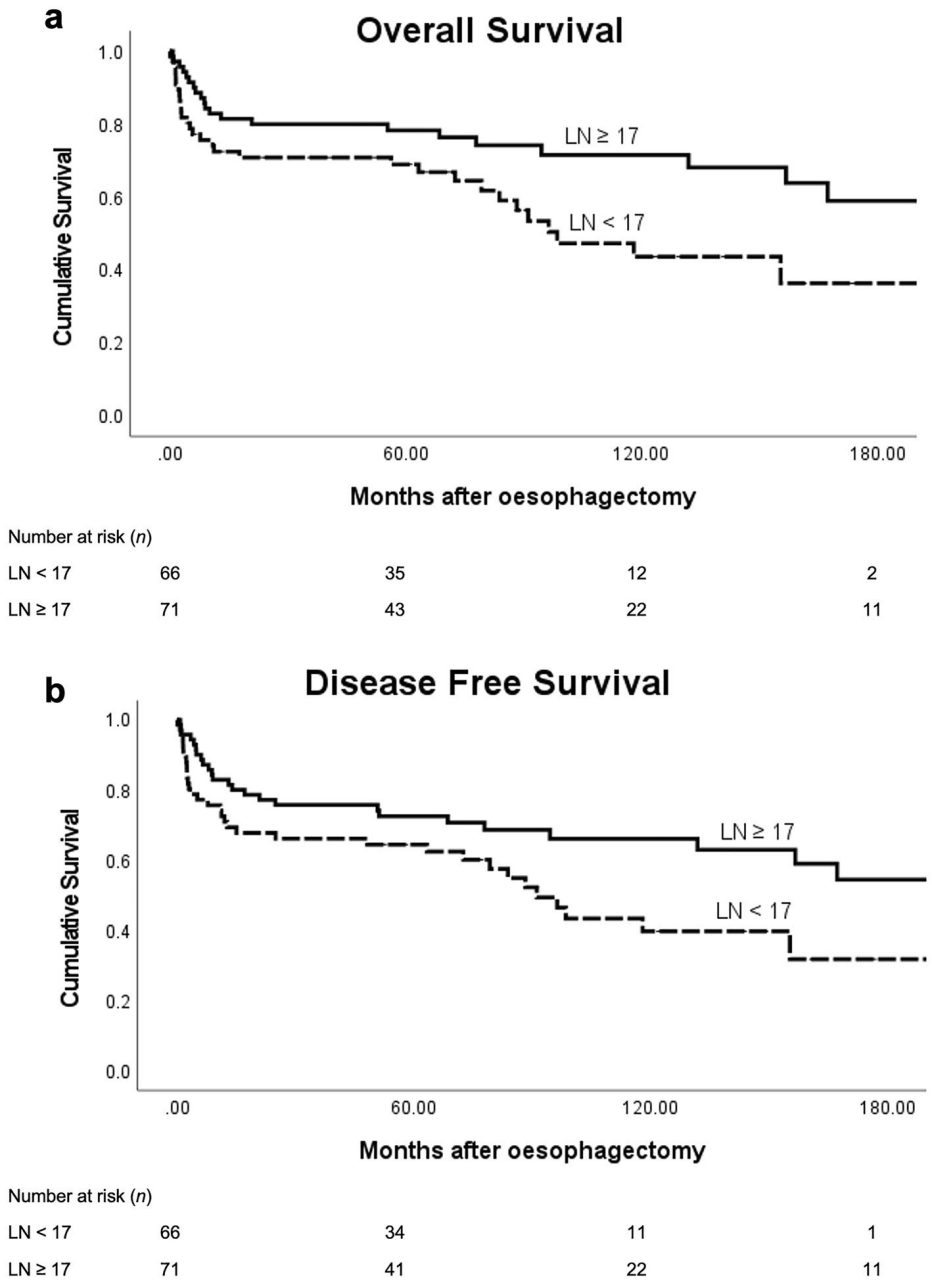
Kaplan–Meier survival curves based on number of resected lymph nodes (LN) in pN0 oesophageal cancer (*n* = 137; 17 or more LNs versus less than 17 LNs). (**a**) Overall survival (Log Rank, *P* = 0.045), (**b**) Disease Free survival (Log Rank, *P* = 0.030). Numbers below graphs indicate the number of patients at risk at each time point for the LN groups.

The median OS was 13.7 years with a 5-year actuarial survival rate of 72%. Kaplan–Meier analysis demonstrated a significant difference in OS (Log Rank, *P* = 0.045, Fig. 1a) and DFS (Log Rank, *P* = 0.030, Fig. 1b) between the lymph node groups (Fig. 1). The median OS and 5-year actuarial survival rates in patients with less than 17 and 17 or more nodes resected was 8.6 years and 67% and 17.3 years and 76% respectively.

**Figure 2:**
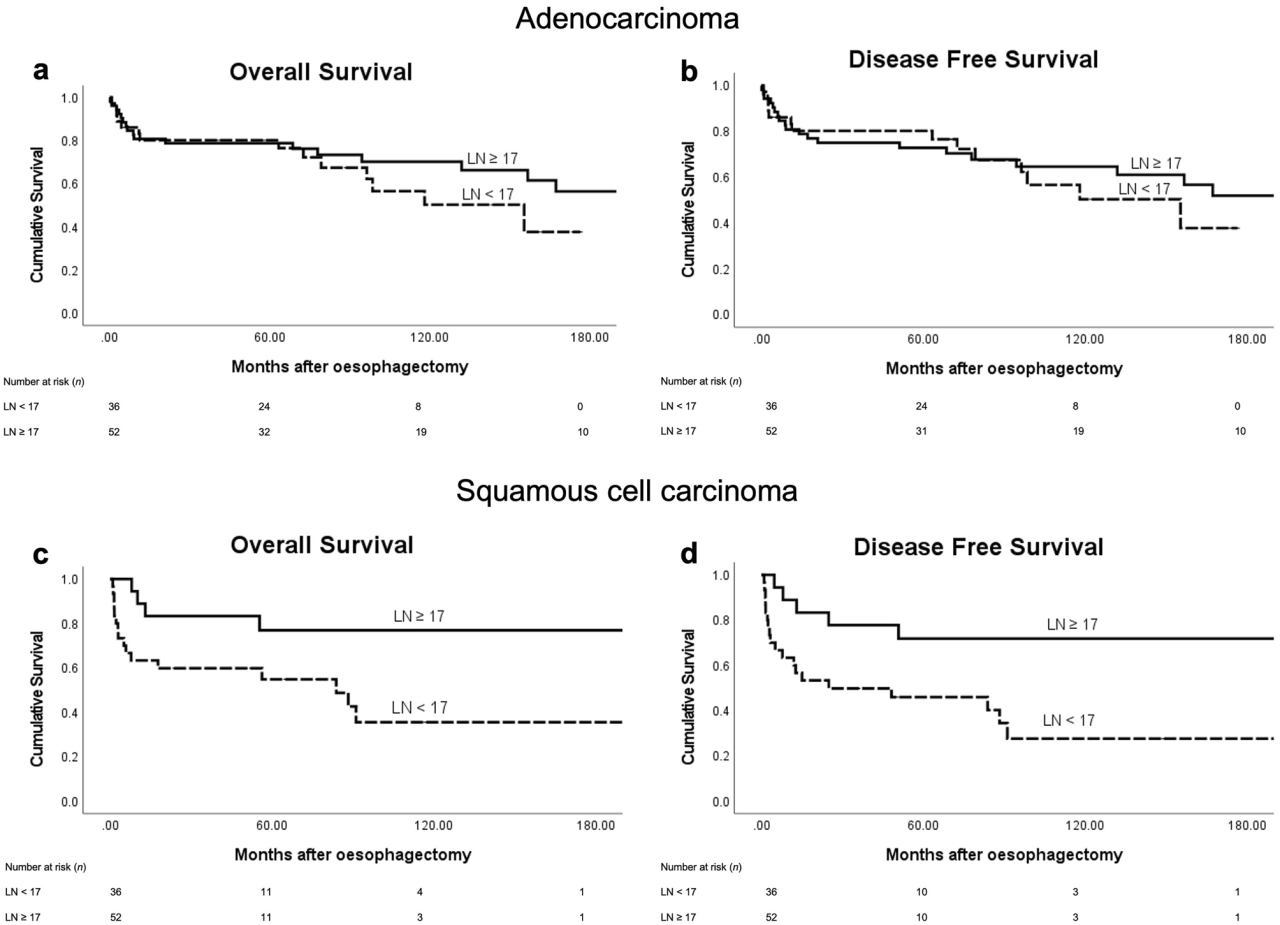
Kaplan–Meier survival curves for histological subtypes (adenocarcinoma, *n* = 88 vs squamous cell carcinoma, *n* = 49) based on number of resected lymph nodes (LN) in pN0 oesophageal cancer (17 or more LNs versus less than 17 LNs). (**a**) Adenocarcinoma—Overall survival (Log Rank, *P* = 0.322), (**b**) Adenocarcinoma—Disease Free survival (Log Rank, *P* = 0.622). (**c**) Squamous cell carcinoma—Overall survival (Log Rank, *P* = 0.047), (**d**) Squamous cell carcinoma—Disease Free survival (Log Rank, *P* = 0.013). Numbers below graphs indicate the number of patients at risk at each time point for the LN groups.

Subgroup analyses for OS and DFS for both histological subtypes were performed (Fig. 2). However, a difference for OS and DFS only reached statistical significance in the SCC group (AC: Log Rank, OS *P* = 0.322, DFS *P* = 0.622 vs SCC: Log Rank, OS *P* = 0.047, DFS *P* = 0.013).

Subgroup analysis for tumour AJCC stage revealed a significant improvement in OS and DFS for stage II patients (*n* = 47) with 17 or more lymph nodes were removed (Log Rank, OS *P* = 0.029, DFS *P* = 0.032). Improvement in OS or DFS based on number of lymph nodes resected for AJCC stages I, III and IV did not reach statistical significance.

Univariate and multivariate hazard ratios (HRs) from Cox regression analysis are shown in Table 2. After adjusting for ten potential confounders (age, sex, tumour histology, location, histological grade, presence of lympho-vascular invasion, stage, neoadjuvant chemotherapy and radiotherapy and adjuvant chemotherapy), a greater number of resected lymph nodes was associated with improved overall survival. In particular, when compared to resecting less than 17 lymph nodes, resecting 17 or more nodes (HR 0.45, 95% CI 0.22–0.92) was associated with a reduced hazard of death. Furthermore, patients with stage IV tumours (3 patients) were independently associated with greater hazard of death (HR 5.98, 95% CI 1.07–33.65).

**Table 2:** Univariate and multivariate predictors of overall survival in pN0 oesophageal cancer.

Characteristic	Univariate analysis		Multivariate analysis	
	HR (95% CI)	*P*-value	HR (95% CI)	*P*-value
Age				
< 65 years	Reference		Reference	
≥ 65 years	0.89 (0.52–1.51)	0.659	0.75 (0.41–1.38)	0.354
Sex				
Female	Reference		Reference	
Male	0.72 (0.41–1.25)	0.238	0.75 (0.35–1.59)	0.447
Histology				
Adenocarcinoma	Reference		Reference	
Squamous cell carcinoma	1.35 (0.77–2.36)	0.294	0.31 (0.10–0.99)	0.048*
Tumour location				
Upper oesophagus	Reference		Reference	
Middle oesophagus	2.21 (0.51–9.69)	0.292	4.76 (0.92–24.61)	0.063
Lower oesophagus	0.66 (0.15–2.94)	0.588	0.95 (0.18–4.87)	0.949
Gastro-oesophageal junction	1.06 (0.25–4.55)	0.940	1.28 (0.25–6.65)	0.772
Histological grade				
G1 (well differentiated)	Reference		Reference	
G2 (moderately differentiated)	1.85 (0.71–4.85)	0.211	1.66 (0.53–5.25)	0.388
G3 (poorly differentiated)	2.35 (0.89–6.21)	0.086	2.28 (0.76–6.80)	0.141
Presence of lymphovascular invasion				
No	Reference		Reference	
Yes	1.06 (0.53–2.12)	0.872	1.32 (0.60–2.91)	0.495
Tumour stage (AJCC8)				
I	Reference		Reference	
II	1.63 (0.90–2.94)	0.105	1.19 (0.59–2.39)	0.635
III	0.74 (0.30–1.83)	0.517	0.36 (0.12–1.10)	0.073
IV	3.54 (0.82–15.26)	0.091	5.98 (1.07–33.56)	0.042*
Number of nodes resected				
< 17	Reference		Reference	
≥ 17	0.58 (0.34–0.99)	0.048*	0.45 (0.22–0.92)	0.028*
Neoadjuvant radiotherapy				
No	Reference		Reference	
Yes	2.40 (1.17–4.92)	0.018*	0.70 (0.21–2.32)	0.564
Neoadjuvant chemotherapy				
No	Reference		Reference	
Yes	1.61 (0.92–2.83)	0.099	3.16 (1.24–8.03)	0.016*
Adjuvant chemotherapy				
No	Reference		Reference	
Yes	0.66 (2.60–1.66)	0.375	0.35 (0.11–1.12)	0.076

*HR* hazard ratio, *CI* confidence interval.

*Significant *P*-value.

Patients who received neoadjuvant radiotherapy had a significantly lower number of lymph nodes harvested than those who did not (mean 8.7 vs 19.4, *U* = 292, *P* < 0.001). Although neoadjuvant radiotherapy was associated with a significantly higher unadjusted hazard of death (HR 2.40, 95% CI 1.17–4.92), after adjusting for potential confounders, it had no effect on overall survival (HR 0.70, 95% CI 0.21–2.32). Interestingly, patients who received neoadjuvant chemotherapy had a significantly higher adjusted hazard of death (HR 3.16, 95% CI 1.24–8.03). However, adjuvant chemotherapy was not an independent predictor of overall survival (HR 0.35, 95% CI 0.11–1.12).

## Discussion

The results of this study highlight the importance of lymphadenectomy in either improving survival in the pathologically node-negative subgroup or upstaging true node positive patients with cancer of the oesophagus.

A key finding in this cohort was that a nodal harvest of 17 or greater was associated with improved survival.

Increased number of lymph nodes in the examined specimen has been shown to increase pathological stage^8^. Incomplete pathologic lymph node examination and under staging patients as a result can falsely worsen stage-per-stage survival. The number of nodes counted in the specimen may reflect the meticulousness of pathological examination and thus improve accuracy of pathological staging, potentially changing stage-based survival.

Whist this difference may be contributed to simply improving accuracy of staging, it is possible that lymphadenectomy improves survival via other pathways. For example, controlling potential micro-metastasis not detected on pathological examination or isolated tumour cell deposits (ITC) which are currently classified as pN0^11^. Thorough lymphadenectomy may offer superior local disease control by removing microscopic deposits possibly not detected by the pathologist. Studies suggested that re-evaluation of lymph nodes with additional immunohistochemistry has been shown to detect occult lymph node disease in up to 17% of patients staged as pN0^12^. Although the effect of ITC in oesophageal carcinoma prognosis remains a subject of debate, it seems more than likely that their prognostic value is similar to that of the micrometastasis^13,14^. It is therefore likely that, for some patients, extensive lymphadenectomy offers improvement in survival by removal of pathologically undetected metastasis or detected ITC (which are reported as pN0).

Neoadjuvant radiotherapy was associated with significantly reduced lymph node count in this study without effect on overall survival. Other studies showed complete pathological response to systemic treatment (in primary tumour and lymph node disease) improves survival, whereas partial or non-responders to neo-adjuvant treatment have significantly worse disease free and overall survival^15,16^. For patients with poor response to neo-adjuvant treatment radical surgery with extensive lymphadenectomy could be the only chance of cure.

Subgroup analysis based on tumour type (AC vs SCC) has shown a trend towards improvement in survival with increased lymph node harvest in both groups, however this difference was only significant in the SCC group. Similar results have been demonstrated in node negative head and neck SCC^17^. Extensive 3-field lymphadenectomy possibly improves survival in node-positive oesophageal SCC^18^. Perhaps lymphadenectomy is even more important in patients with SCC possibly offering better disease control to that of radiotherapy?

Survival benefit was most pronounced in patients with stage II disease. This significant difference in OS and DFS may support the hypothesis of increasing stage accuracy with increased nodal count most evident in patients with T2 and T3 tumours (stage II). It may be most noticeable in this group as the node status is the stronger determinant of survival in patients with larger tumours. Lymph node involvement can be an indicator of increased risk of distant metastasis which account for significant disease specific mortality in oesophageal cancer.

Management of regional lymph nodes appears to play a key role in improving outcomes of patients with oesophageal cancer^19^. Future research should be directed in risk stratifying patients based on molecular classifiers^20^. This may allow less invasive treatment in some patients with early tumours (T1) and those having complete clinical response to neoadjuvant treatment. Until the accuracy of non-invasive methods of assessing for complete clinical response improve, surgery with complete lymphadenectomy (upfront or salvage) remains standard treatment^21,22^. The concept of a sentinel lymph node has been proven in oesophageal cancer^23^. It is possible that in the future endoscopic mucosal resection and sentinel lymph node biopsy can become a standard of care in select complete responders and T1b patients to reduce the morbidity of oesophagectomy. Although the relatively high rate of skip nodal metastasis remains of concern. Data in this study suggested extended lymphadenectomy remains the gold standard for curability and lesser lymphadenectomy may risk reducing survival. This study supports current NCCN recommendation in minimum of 15 lymph node harvest for all patients undergoing oesophagectomy for cancer even if clinically staged as N0 and suggests that in pN0 subgroup removing even more nodes (≥ 17 in this group) can be beneficial^2^.

Active surveillance of SCC with clinical complete response and salvage surgery in recurrent disease has been proposed by some authors with promising results^22^. Further research is needed to assess whether survival benefit of removal of ≥ 15 nodes is sustained in patients undergoing salvage surgery where lymph node count is expected to be lower.

The strengths of this study include single surgeon database allowing for a standardised procedure and treatment protocols that reflect current management (supporting generalisability), and robust prospective data collection. The small number of patients receiving neoadjuvant treatment in this cohort limits bias of effects of systemic treatment by complete pathologic response. The main limitations of this study are retrospective cohort design, and the lack of data on circumferential resection margin.

## Conclusion

In this cohort of patients with pN0 oesophageal cancer lymph node count of ≥ 17 was associated with improved overall survival and disease-free survival. Possible explanations for this include improved staging accuracy and improved local disease control and until this can be quantified, radical lymphadenectomy will remain the treatment of choice.

## Data Availability

The data that support the findings of this study are available from the corresponding author upon reasonable request. Raw data can be made available on request.
